# Profile of Bacterial Infections in COVID-19 Patients: Antimicrobial Resistance in the Time of SARS-CoV-2

**DOI:** 10.3390/biology10090822

**Published:** 2021-08-24

**Authors:** Irene Stefanini, Giuseppe De Renzi, Elisa Foddai, Elisa Cordani, Barbara Mognetti

**Affiliations:** 1 Department of Life Sciences and Systems Biology, University of Turin, Via Accademia Albertina 13, 10123 Turin, Italy; irene.stefanini@unito.it; 2 SCDO Laboratory of Clinical Pathology and Microbiology, San Luigi Gonzaga University Hospital, Regione Gonzole 10, Orbassano, 10043 Turin, Italy; g.derenzi@sanluigi.piemonte.it (G.D.R.); elisa.foddai@gmail.com (E.F.); elisa.cordani@gmail.com (E.C.)

**Keywords:** bacterial infections, AMR, SARS-CoV-2, COVID-19

## Abstract

**Simple Summary:**

Since the beginning of COVID-19 pandemic, no specific drugs have been available to treat the SARS-CoV-2 infection, therefore antibiotics have been often used both for prophylactic and therapeutic purposes. Their wide use, though, is known to contribute to the emergence of antimicrobial resistance. Aiming at evaluating the impact of the COVID-19 pandemic on the distribution and characteristics of bacterial infections, and on the frequency of antimicrobial resistance, we investigated the microbial strains identified through laboratory tests on clinical specimens from COVID-19 and non-COVID-19 patients accessing an Italian tertiary hospital over nearly one year. We highlighted that COVID+ patients bore a significantly higher number of bacterial species. Eight out of the 100 species identified were isolated exclusively from COVID+ and most of them are known to establish infections only in immunocompromised patients. Resistance to every tested antibiotic was seen in 8.3% of the isolates with a correlation with the positivity to COVID, but neither all COVID+ or COVID− isolates showed characteristic responses to the tested antibiotics. The predicted increase of antibiotic resistance is not observable yet, but the higher frequency of multi-resistant COVID+ isolates suggests that it is actually occurring, further calling for the definition of alternative treatments of COVID-19 infections.

**Abstract:**

The global onset of severe acute respiratory syndrome coronavirus 2 (SARS-CoV-2) virus infections happened suddenly, hence imposing a rapid definition of effective therapeutic approaches. Antibiotics were included among the prophylactic agents because of both the similarity between SARS-CoV-2 and atypical pneumonia symptoms, and the immune-modulating and anti-inflammatory properties of such drugs. Although, this approach could exacerbate the emergence of antimicrobial resistance. To evaluate the impact of the COVID-19 pandemic on the spread and characteristics of bacterial infections, as well as on the frequency of antimicrobial resistance, we investigated and compared clinical bacterial strains isolated in an Italian hospital from COVID-19 patients and non-COVID-19 patients during and before the COVID-19 outbreak. Data clearly indicate the impact of the COVID-19 pandemic on bacterial infections: not only some bacterial species were found in either COVID-19 positive or in COVID-19 negative patients, but isolates from COVID-19 patients also showed higher levels of antimicrobial resistance. Nevertheless, despite some bacterial species were isolated only before or over the pandemic, no differences were observed among the antimicrobial resistance levels. Overall, these results recapitulate the current situation of microbial infections and could also provide an overview of the impact of COVID-19 on bacterial pathogens spread and resistance.

## 1. Introduction

The sudden, massive, and rapid spread of the pandemic caused by the severe acute respiratory syndrome coronavirus 2 (SARS-CoV-2) virus (COVID-19) has demanded the adoption of drastic actions to contain the further spread of the infection and to rapidly adjust healthcare systems and frameworks. In Italy, where the first domestic case was detected on 28 February 2020, early epidemic phases caught the National Healthcare System unprepared for such an event and a severe health crisis was averted by a rigid lockdown from 9 March to 3 May 2020, followed by a period of mitigation. Unfortunately, new restrictive measures had to be reintroduced in November 2020 due to the increase in the number of cases [1]. In the first wave (from 21 February 2020 to 11 June 2020), the total number of diagnosed cases was 233,019 and the total number of deaths was 34,260, while in the shorter period of the second wave (from 14 September 2020 to 31 December 2020) 409,241 cases were diagnosed and 38,535 people died. Southern Italian regions, which were preserved by the first wave, were the most affected during the second one [2]. Considering the urgency to alleviate the symptoms and resolve the infection, treatments are being experimentally defined [3]. The symptoms of COVID-19 infection are highly similar to atypical bacterial pneumonia [4,5,6] and this lead to the empirical decision to administer antimicrobials commonly used for the treatment of bacterial/fungal pneumonia in 72% of cases, worldwide, either to prevent the onset of concurrent infections or to exacerbate already present concomitant bacterial or fungal infections, despite microbial co-infections being observed only in 8% of COVID-infected patients [7]. Furthermore, antibiotics could be useful in the treatment of COVID-19 positive patients because of their immune-modulating, anti-inflammatory, and potential antiviral properties [8] has to be considered, however, the antiviral effectiveness of some antibiotics (aminoglycosides and meropenem) [9,10] has not been proven yet or has been proven to be limited (fluoroquinolones) [11].

As a consequence, despite the indications of scientific societies and working groups, antibiotics were over-prescribed and administered, particularly during the first wave of the pandemic in Italy. Early indications provided by scientific societies and working groups suggesting to limit the administration of targeted antibiotics only in the presence of a reasonable suspicion of concomitant bacterial or viral infection [12] were later confirmed by SIMIT (Italian Society of Infectious and Tropical Diseases) (November 2020) [13] and SIMG (Italian society of general medicine) (April 2021) [14].

In addition to the clinical oculate decision of prescribing antibiotics for the treatment of COVID-19 infections, an online survey carried out in the Australia at the height of the initial outbreak has revealed that almost 20% of participants deliberately decided to take antibiotics as a preventive measure against COVID-19 infections [15].

Together, these factors may contribute to further exacerbating the emergence of antimicrobial resistance [16,17,18], and the World Health Organization (WHO), to prevent this, issued guidance to discourage antibiotic therapy or prophylaxis for patients with mild or moderate COVID-19 symptoms unless there is a clinical indication of a bacterial infection [19].

Aiming at evaluating the impact of the COVID-19 pandemic on the distribution and characteristics of bacterial infections, as well as on the frequency of antimicrobial resistance, we investigated and compared the characteristics of microbial strains identified through laboratory tests on clinical specimens from COVID-19 patients and non-COVID-19 patients.

## 2. Materials and Methods

### 2.1. Isolation of Strains

The information on the isolates collected in the period between 26 March 2020 and 9 January 2021 and analyzed over this study was collected at the San Luigi Hospital in Turin (Italy) (Supplementary Table S1). The sources of isolation were: articular liquid, ascites, aspirated bronchus, bile, biopsy from lymph node, biopsy from other body sites, blood, bronchial lavage, catheter, catheter urine, cavitary liquid, cutaneous lesion, drainage liquid, exudate, feces, liquor, nasal tampon, oral tampon, peritoneal liquid, pharynx, bedsore, pleura liquid, prostatic material, pus, rectal tampon, sputum, surgical wound, ureter, urethra secretion, urine, vaginal secretion, venous catheter, and “others” (not specified in the medical report). For statistical reasons we grouped the clinical wards according to the corresponding clinical area: cardiology, ER, ICU (anesthesiology and reanimation), medicine (diabetology, geriatrics, internal medicine, hematology, low intensity COVID, medicine and surgery, neurology, oncology, pneumology, rehabilitation), surgery (general surgery, orthopedics, otolaryngology, thoracic surgery, urology) (Supplementary Table S1). Neither clinical nor demographic data were collected for patients from which the strains were isolated. Hence, Ethical Committee approval is not required. Bacteria isolation was carried out by seeding the samples onto selective media (VACUTEST KIMA and BIOMERIEUX ITALIA). Bacteria were identified at the genus or species level through the biochemical BD Phoenix™ (Becton Dickinson, Milan, Italy) system, which is based on 45 biochemical reactions whose outcomes, taken together, allow the identification of the microorganism.

### 2.2. Antimicrobial Testing

To establish the susceptibility of the bacterial isolates under investigation to the most commonly administered antibiotics, the antibiogram was obtained for each isolate according to the ISO and EUCAST Broth microdilution method. Briefly, the antibiogram was performed by inoculating 5 × 10^5^ CFU/mL bacterial cells in MH-F broth (Mueller Hinton broth supplemented with 5% lysed horse blood and 20 mg/L β-NAD), supplemented with antibiotic in multi-well plates and incubated at 35 °C for between 16 and 48 h (depending on the species), after which plates were visually inspected to determine the MIC (minimum inhibitory concentration) of the antibiotic against the tested microorganism. The tested concentrations of each antibiotic were chosen to include at least twice the highest clinical breakpoint concentration and half of the lowest breakpoint concentration. For each species, a different set of antibiotics was tested, as reported in Supplementary Table S2. Each isolate was scored as R (Resistant), I (Intermediate), or S (Susceptible) to each tested antibiotic according to the clinical breakpoints defined by EUCAST for the corresponding species and antibiotics and reported in the clinical breakpoint table v7.0 [20].

### 2.3. Statistical Analyses

Provided that we did not have access to clinical and demographic data of the patients from which the strains were isolated, the relevance of such variables on the statistical analyses cannot be assessed. For alpha and beta diversities estimation and comparison, isolates were grouped as being isolated on the same date (month and year), from the same specimen, and from patients with the same COVID-19 status (infected or not). With this grouping method, isolates from the same patient would be likely pooled together (hence erroneously considered independent). To assess whether this grouping led to biased results, all the statistical tests described afterwards have been carried out also on samples grouped by patient, obtaining the same results as those obtained on the samples grouped as previously described. Hence, the pooling of isolates did have a minor impact, if any, on the analyses. Alpha (within-sample richness,) and beta-diversity (between-sample dissimilarity) estimates were computed using the phyloseq R package [21]. Briefly, whereas alpha diversity indexes (observed, Shannon, or Simpson) summarize the structure of an ecological community (in this case a group of isolates) with respect to its richness (in this case the number of species), evenness (distribution of abundances of the species), or both, beta diversity is the difference in diversity of species between two or more group of isolates, expressed as the total number of species that are shared or unique to each of the groups being compared [22]. Principal coordinate analysis was carried out by using the function *ordinate()* in the phyloseq library [21] on Jaccard distances calculated on the composition of microbial communities in groups of isolates (composed as described earlier). To assess the differences among groups, permutational multivariate analysis of variance (permanova) was performed using the *adonis()* function of the vegan R package with 999 permutations [23]. Two-sided, unpaired Welch *t*-statistics were computed using the function *mt()* in the phyloseq library [21], and the *p*-values were adjusted for multiple comparisons controlling the family-wise Type I error rate (minP procedure) [24]. Unpaired two-samples Mann-Whitney U test (Wilcoxon test) statistics were computed using the function *wilcox.test()* in the stats library [25] and the *p*-values were adjusted for multiple comparisons by computing false discovery rate (FDR)-adjusted *p*-values using the Benjamini-Hochberg procedure [26]. For each tested antibiotic, resistance percentage was calculated as the number of resistant isolates over the total isolates tested with the corresponding antibiotic. Fisher exact test was carried out to assess the enrichment of any isolation sources among the isolates from COVID+ and COVID− patients showing significantly different levels of susceptibilities to the tested antibiotics. To evaluate the impact of different typology of COVID-19 cases and therapeutic approaches on the insurgence of (resistant) bacterial infections, alpha and beta diversities, as well as the bacterial species were compared between samples isolated over the first and the second pandemic Italian waves, which lasted from March to September 2020 and from October 2020 to January 2021 respectively.

All figures were created using R. All diagrams were created using R and combined as panels with the Inkscape software.

## 3. Results

### 3.1. Isolation of Bacterial Strains from Clinical Specimens Collected from COVID+ and COVID− Patients

**Figure 1:**
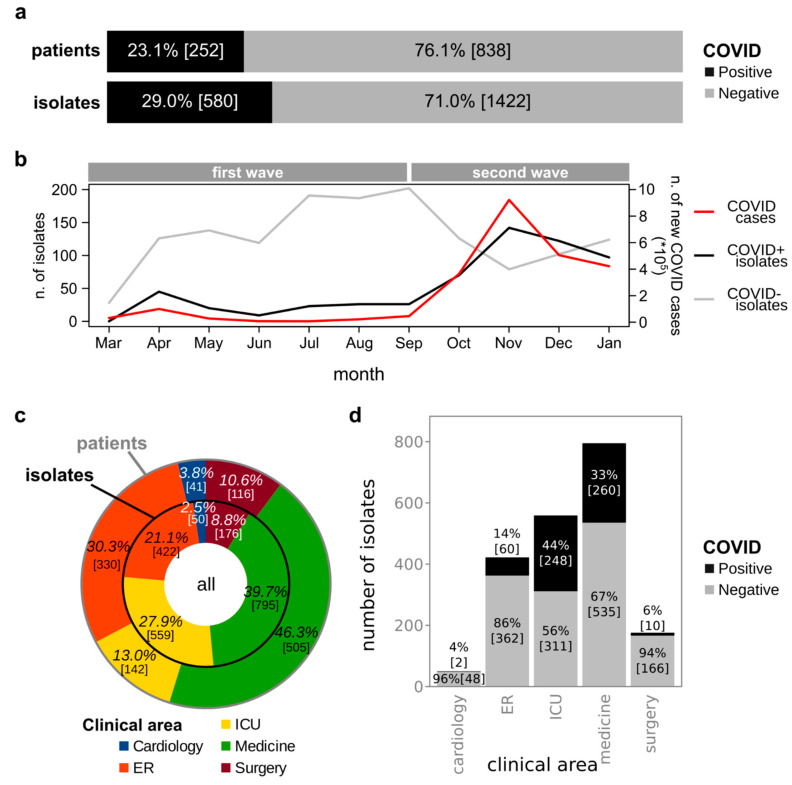
Distribution of samples among patients having been tested positive (COVID+) or negative (COVID−) for COVID-19 before collection of samples for microbial analysis. (**a**) Percentages and number of patients (and relative isolates) having been tested positive or negative for COVID-19 before collection of samples for microbial analysis. (**b**) Comparison of trends of new COVID-19 cases in Italy (red line, right vertical axis), bacterial strains isolated from COVID+ patients in our study cohort (black line, left vertical axis), and bacterial strains isolated from COVID− patients in our study cohort (grey line, left vertical axis). The time-periods of the first and second pandemic waves in Italy are reported over the plot. (**c**) Distribution of patients and isolates among clinical areas. (**d**) Distribution of isolates from COVID+ and COVID− patients among clinical areas.

In a period of almost one year (between March 2020 and January 2021), a total of 2002 bacterial strains were isolated from 1090 patients symptomatic of bacterial infections, identified, and characterized at the San Luigi Hospital in Turin (Italy) (Supplementary Table S1). Among these isolates, 28.97% (*n* = 580) were isolated from 252 patients (23.1% of all the analyzed patients) having been tested positive for COVID-19 at the time of samples collection (COVID+), the remaining 1422 strains (76.9%) were isolated from 838 COVID-19 negative (COVID−) patients (Figure 1a). Notably, the number of bacterial strains isolated per month from COVID+ patients did correlate with the number of new COVID-19 patients observed at the national level [27] (Pearson r = 0.96, *p* = 4.62 × 10^−6^, Figure 1b). Contrarily, the number of bacterial strains isolated per month from COVID− patients did not correlate with the number of new COVID-19 patients (Pearson r = −0.41, *p* = 0.214) nor with the number of bacterial strains isolated from COVID+ patients (Pearson r = −0.26, *p* = 0.436) (Figure 1b). Despite the number of patients from which strains were isolated was greater over the first time-periods than over the second time-periods (respectively 648 and 478, with 36 patients being included in both groups), the average number of patients analyzed per month was greater over the second time-periods (119.5 vs. 92.57 average patients per month over the first period). Furthermore, whereas only the 10.4% of the patients analyzed over the first period were COVID+, the 41.2% of patients analyzed over the second period were COVID+, in line with the peak of new COVID-19 cases observed at the national level over the second period (with a peak of 40,902 new cases on the 13 November 2020) compared to the first period (with a peak of 6203 new cases on the 26 March 2020). The analysis of specimens from 406 patients resulted in the isolation of multiple isolates belonging to different species. Conversely, the microbiological analysis on the remaining 684 patients resulted in the isolation of a single strain. The COVID-19 status of the patient (having been tested positive or negative for COVID) and the isolation of multiple or single strains from the analyzed specimen were not independent (Chi-square test, *Χ*^2^(1, N = 1090) = 7.3, *p* = 0.007). In particular, 64.9% and 55.5% of specimens from COVID-19 negative and positive patients respectively resulted in the isolation of a single strain (Supplementary Figure S1). The 44.5% of COVID+ patients provide multiple isolates, which, compared to the 35.1% of COVID− patients resulting in the isolation of multiple strains, indicated that COVID+ patients had a higher prevalence of multiple strains. Our dataset included patients hospitalized in five different clinical areas: cardiology, ER (Emergency Room), ICU (Intensive Care Unit), medicine, and surgery (Figure 1c). Some patients were hospitalized in multiple clinical areas (Supplementary Figure S2a). Medicine was the clinical area where almost half of the patients accessed (*n* = 505 out of 1090 patients, 46.3%), followed by the ER (*n* = 330, 30.3%), the ICU (*n* = 142, 13.0%), surgery (*n* = 111, 10.6%), and cardiology (*n* = 41, 3.8%) (Figure 1c). Notably, the same order was not confirmed when considering the number of isolates: whereas medicine was again the clinical area from which the highest number of strains were isolated (*n* = 795, 44.5%), the second and third place were switched, with ICU providing the second-highest number of isolates (*n* = 559, 27.9%), and the ER being the third (*n* = 422, 21.2%) (Figure 1c). In fact, the number of patients hospitalized in the different clinical areas did not correlate with the number of strains isolated from patients hospitalized in the corresponding clinical area (Pearson *p* = 0.083). It is worth noting that, whereas the average number of isolates per patient hospitalized in cardiology, in the ER, in medicine, and in surgery ranged between 1.2 and 1.5 (respectively: 1.2, 1.3, 1.5, 1.5), the specimens collected from patients hospitalized in the ICU provided a higher average of isolates (3.9). The same trend was observed when considering the entire set of isolates and when considering separately the isolates from COVID+ or COVID− patients: the average number of isolates per patient was approximately 2 for both subsets of samples (Supplementary Figure S2b,c).

A significant relationship was found between the number of COVID+ and COVID− patients in different clinical areas and the number of isolates per clinical area (Chi-square test, *Χ*^2^ (1, N = 2002) = 7.3, *p* = 0.007, Figure 1d). However, the number of isolates per COVID+ and COVID− patients did not differ in any clinical area (Wilcoxon-Mann-Whitney *p* > 0.05, Supplementary Figure S3). Conversely, the number of isolates per patient in the ICU was significantly higher than the number of isolates per patient in cardiology, the ER, medicine, and surgery (Supplementary Figure S3). Furthermore, the number of isolates per patient in the ER was lower than the number of isolates per patient in medicine and surgery (Supplementary Figure S3). In addition, some significant differences were also observed among the number of isolates per patient when analyzing separately isolates from COVID+ and COVID− patients. In fact, COVID− patients in the ER showed a lower number of isolates compared to those in the ICU, medicine, and surgery; COVID− patients in cardiology bore fewer strains compared to COVID− patients in the ICU, medicine, and surgery; COVID− patients in the ICU showed more isolates than those in medicine and surgery. Conversely, COVID+ patients in the ICU bore more strains compared to those in medicine and surgery (Supplementary Figure S3). Concerning the source of isolation of the strains, there were no specimens significantly more analyzed in COVID+ or in COVID− patients (Supplementary Figure S4). Together, these data suggest that ICU patients, either COVID+ or COVID−, have a higher chance of multi-strains infections and that multi-strain infections are more frequently detected in long-term clinical areas (the ICU, medicine, and surgery) than in the ER, with the latter more likely coping with community-acquired infections.

### 3.2. Microbial Species Isolated from COVID+ and COVID− Patients

**Figure 2:**
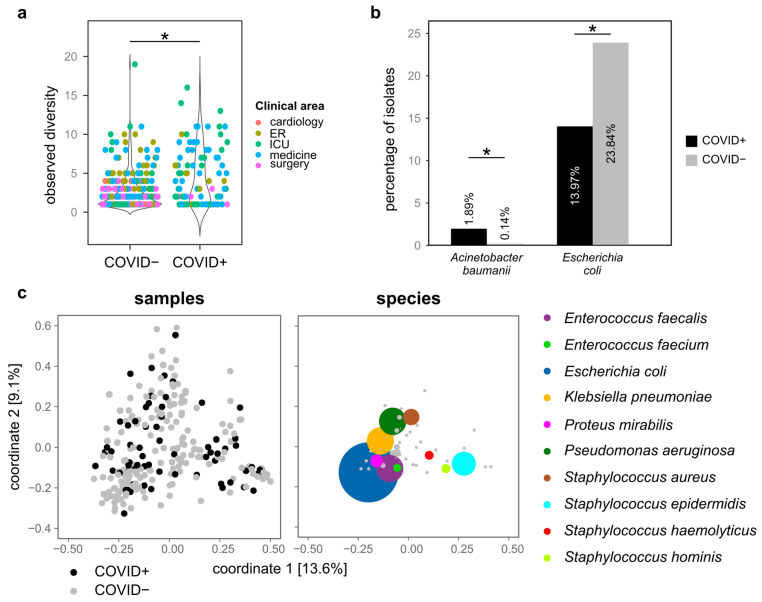
Distribution of bacterial species among patients. (**a**) Observed number of species per patient, grouped according to the COVID-19 status. Each point indicates a different group of strains (grouped as being isolated in the same month and year, from patients hospitalized in the same clinical area and being either COVID+ or COVID−). (**b**) Species whose abundance significantly differed among COVID+ and COVID− patients. (**c**) First two coordinates of the principal coordinate analysis based on the Jaccard distances calculated among samples grouped as described for panel a. On the left part, the distribution of isolates, on the right part the distribution of the most abundant bacterial species. * = Wilcoxon-Mann-Whitney *p* value < 0.05.

We then focused on the distribution of bacterial species, first by comparing the number (measured as observed, Shannon, and Simpson alpha diversity indexes) of species isolated from samples grouped according to the year or month of isolation, COVID-19 status (COVID+ or COVID−), clinical area, and source of isolation, then by assessing the presence of species isolated uniquely to either COVID+ or COVID− patients. The strains under investigation belonged to a total of 100 species, distributed among 33 genera (Supplementary Table S3). The most abundant species was *Escherichia coli* (420 isolates), followed by *Klebsiella pneumoniae* (*n* = 192), *Pseudomonas aeruginosa* (*n* = 187), *Enterococcus faecalis* (*n* = 184), *Staphylococcus epidermidis* (*n* = 175), and *Staphylococcus aureus* (*n* = 134) (Supplementary Table S3). The remaining species were represented with less than 100 isolates, and 42 species showed only one isolate each (Supplementary Table S3). According to every compared alpha diversity index, COVID+ patients bore a significantly higher number of bacterial species compared to COVID− (Figure 2a and Supplementary Figure S5a, Wilcoxon-Mann-Whitney *p* < 0.05). Similarly, a significantly lower number of species was isolated from patients hospitalized in the surgery area compared to the patients in the ER, ICU, and medicine and in cardiology compared to medicine and the ER (Supplementary Figure S5b). Furthermore, bronchoaspiration, blood, catheter urine, and venous catheter were the specimens associated with the highest number of bacterial species (Supplementary Figure S5c and Supplementary Table S4). Contrarily, we did not observe significant differences among the alpha diversity indexes of bacterial sub-populations isolated from the different patients nor from COVID+ or COVID− patients analyzed over the first or second waves of the pandemic in Italy.

Among the 100 identified species, 8 were isolated only from COVID+ patients, 49 were isolated only from COVID− patients, and the remaining 45 were present in both COVID+ and COVID− specimens (Supplementary Table S5). To note, most of the species isolated only from either COVID+ or COVID− patients can be considered accidental, as these species were represented by only one isolate (7 out of the 8 species isolated only from COVID+ patients and 33 out of the 49 species isolated only from COVID− patients, Supplementary Table S5). Conversely, the species isolated repeatedly only from either COVID+ or COVID− patients were: *Corynebacterium* spp. (3 isolates from COVID+ patients), *Achromobacter* spp. (*n* = 7, COVID−), *Citrobacter youngae* (*n* = 2, COVID−), *Corynebacterium matruchotii* (*n* = 3, COVID−), *Enterobacter asburiae* (*n* = 2, COVID−), *Hafnia alvei* (*n* = 4, COVID−), Mycobacterium tuberculosis complex (*n* = 2, COVID−), *Serratia liquefaciens* (*n* = 2, COVID−), *Staphylococcus* coag. negative (*n* = 5, COVID−), *Staphylococcus cohnii* spp. urealyticum (*n* = 2, COVID−), *Staphylococcus schleiferi* (*n* = 2, COVID−), *Staphylococcus xylosus* (*n* = 2, COVID−), *Streptococcus agalactiae* Gr B (*n* = 3, COVID−), *Streptococcus bovis* II (*n* = 2, COVID−), *Streptococcus dysgalactiae* spp. *equisimilis* (*n* = 3, COVID−), *Streptococcus oralis* (*n* = 2, COVID−), *Streptococcus pyogenes* Gr A (*n* = 2, COVID−) (Supplementary Table S5). To note, whereas the species identified only among COVID− isolates are commonly considered as opportunistic pathogens capable of setting human infections, most of the species found only among COVID+ strains are known to establish infections only in immunocompromised patients (2 out of the 8 COVID+ specific species vs. 3 out of the 49 COVID− specific species, Supplementary Table S5). Furthermore, two species were significantly more frequently isolated from either COVID+ or COVID− specimens: whereas *Acinetobacter baumannii* isolates were more abundant among COVID+ isolates than among COVID− isolates (1.89% of COVID+ isolates, 0.14% of COVID− strains), *Escherichia coli* was more frequently isolated from COVID− patients (23.84% and 13.97% of the strains isolated from COVID− and COVID+ patients, respectively) (Wilcoxon-Mann-Whitney *p* < 0.05, Figure 2b).

Despite the presence of bacterial species characterizing COVID+ and COVID− patients, these characteristics were not sufficient to determine bacterial population profiles characterizing these two groups of patients. Indeed, as clearly depicted by the first two components of the principal coordinates analysis on Jaccard distances among groups of isolates (Figure 2c), the composition of bacterial populations was not statistically different between COVID+ and COVID− patients (permutational multivariate analysis of variance *p* = 0.131). Similarly, there was not a significant difference in the microbial composition of sub-populations isolated from the different patients nor from COVID+ or COVID− patients analyzed over the first or second waves of the pandemic in Italy.

### 3.3. Antimicrobial Susceptibility of Bacterial Strains Isolated from COVID+ and COVID− Patients

**Figure 3:**
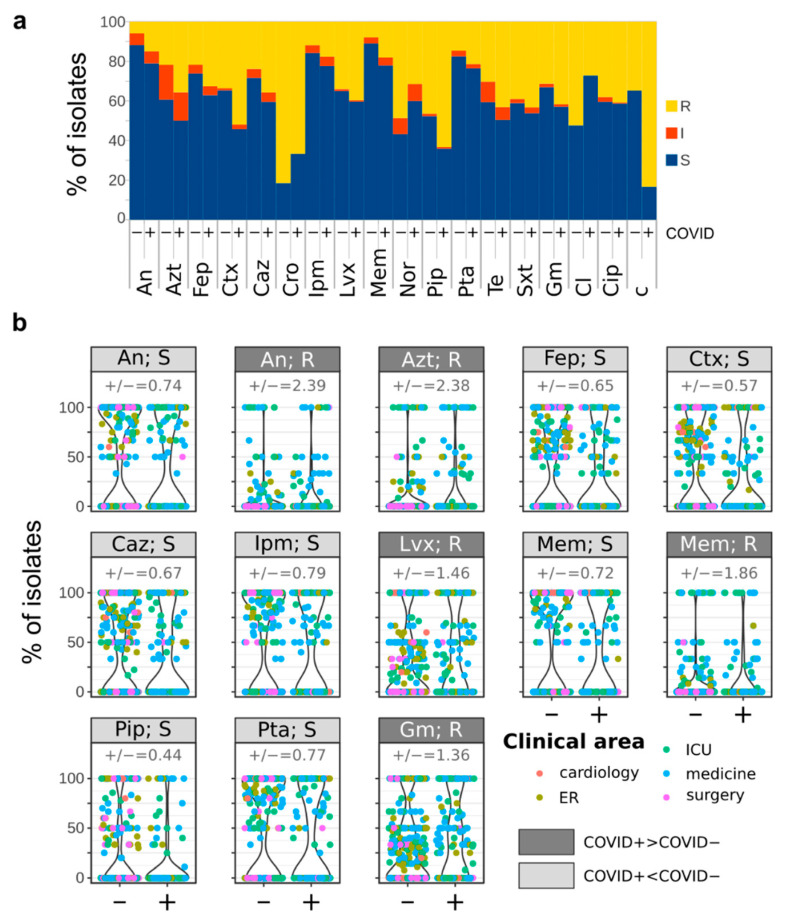
Distribution of antimicrobial resistances among COVID+ and COVID− patients. (**a**) percentages of isolates Resistant (R), Intermediate (I), and Susceptible (S) to the indicated antibiotics. (**b**) Violin plots showing the responses to antibiotics significantly different between COVID+ and COVID− patients’ isolates (Wilcoxon-Mann-Whitney *p* < 0.05). “+/−” = ratio between the percentage of COVID+ and COVID− isolates showing the corresponding response to the antibiotic. An = amikacin, Azt = aztreonam, Fep = cefepime, Ctx = cefotaxime, Caz = ceftazidime, Cro = ceftriaxone, Ipm = imipenem, Lvx = levofloxacin, Mem = meropenem, Nor = norfloxacin, Pip = piperacillin, Pta = piperacillin-tazobactam, Te = tetracycline, Sxt = trimethoprim/sulfamethoxazole, Gm = gentamicin, Cl = colistin, Cip = ciprofloxacin, c = chloramphenicol.

The susceptibility of bacterial isolates was assessed for a total of 18 antibiotics, with the set of antibiotics tested depending on the species of the isolate under investigation (Supplementary Table S2). The antibiotic that was tested against the largest number of isolates was ciprofloxacin (Cip, *n* = 1828), a fluoroquinolone with broad-spectrum bactericidal activity, followed by gentamicin (Gm, *n* = 1788) and trimethoprim/sulfamethoxazole (Sxt, *n* = 1761). The antibiotic showing the highest percentage of resistant isolates (77.8%, *n* = 28) was ceftriaxone (Cro), whereas the antibiotics showing the lowest percentages of resistant isolates were amikacin (An, 8.7%, *n* = 89), meropenem (Mem, 10.6%, *n* = 37), imipenem (Ipm, 13.4%, *n* = 52), and piperacillin-tazobactam (Pta, 16.5%, *n* = 189) (Supplementary Table S2). One hundred and sixty-six strains, corresponding to 8.3% of the isolates, were resistant to every tested antibiotic (Supplementary Table S6). Among these, 72 were isolated from COVID+ patients (12% of the strains isolated from COVID+ patients) and 94 were isolated from COVID− patients (6.6% of the strains isolated from COVID− patients), indicating a correlation between the positivity to COVID-19 and the resistance to every tested antibiotic (Chi square test, *Χ*^2^ (1, N = 2002) = 18.2, *p* = 0.000019). We found correlations between the percentage of strains isolated over the analyzed months from either COVID+ or COVID− patients and resistant to some antibiotics and the number of new COVID-19 cases at the national level in the same month (Supplementary Figure S6). In particular, we found that the number of new COVID-19 cases was negatively correlated with the resistances to colistin (Cl) of both COVID+ and COVID− isolates, positively correlated with the resistances to norfloxacin (Nor), tetracycline (Te), and chloramphenicol (c) of both COVID+ and COVID− isolates, negatively correlated with the resistance of COVID− isolates to cefepime (Fep), ceftazidime (Caz), ceftriaxone (Cro), and imipenem (Ipm), and positively correlated with the resistance of COVID− isolates to piperacillin (Pip) (Supplementary Figure S6). To note, the single *Corynebacterium renale*, *Corynebacterium jeikeium*, *Enterococcus avium*, and *Burkholderia cepacia*, all the *Corynebacterium matruchotii* (*n* = 3, all from COVID+ patients), and the vast majority of *Enterococcus faecium* (41 out of 52 total isolates, corresponding to the 78.8%) and *Corynebacterium amycolatum* (3 out of 4 total isolates) isolates were resistant to all the tested antibiotics (Supplementary Table S5 and Table S6). Although neither all COVID+ or COVID− isolates showed characteristic responses to the tested antibiotics (e.g., all the COVID+ isolates being resistant to a given antibiotic, Figure 3a), significant differences were observed between the responses of COVID+ and COVID− isolates to some antibiotics (Figure 3b). In particular, strains isolated from COVID+ patients were significantly more resistant to amikacin (An), aztreonam (Azt), levofloxacin (Lvx), meropenem (Mem), and gentamicin (Gm) compared to COVID− isolates (Wilcoxon-Mann-Whitney *p* < 0.05, Figure 3b). Conversely, COVID− isolates were significantly more susceptible to cefepime (Fep), cefotaxime (Ctx), ceftazidime (Caz), imipenem (Ipm), piperacillin (Pip), and piperacillin-tazobactam (Pta) (Wilcoxon-Mann-Whitney *p* < 0.05, Figure 3b).

### 3.4. Comparison of Bacterial Infections before and during the COVID-19 Pandemic

Considering the suspected impact of COVID-19 treatments on AMR (antimicrobial resistance), we wondered if this hypothesized AMR increase could be (already) detected. Hence, by taking advantage of a dataset including information on bacterial strains isolated from the same Hospital prior to the onset of the pandemic (2018–2019) [28], we compared the species and relative antibiotic resistances of strains isolated from clinical patients before and during the COVID-19 pandemic from both COVID+ and COVID− patients. The dataset of the pre-pandemic survey encompassed 1583 bacterial isolates belonging to 89 species distributed in 33 genera and 24 families. Whereas sixty-three species were largely represented in the dataset (with at least 5 isolates), 28 species (21 different genera) were represented by only one isolate.

Whereas 61 bacterial species were consistently isolated both before and during the COVID-19 pandemic (without differences in the frequency of isolation, Supplementary Table S7), 28 species were isolated only before the advent of the COVID-19 pandemic (Supplementary Table S7). However, it has to be noted that for only 11 species out of these 28 more than one strain had been isolated before COVID-19: *Bacteroides fragilis* (*n* = 2), *Klebsiella pneumoniae* spp. *ozaenae* (*n* = 3), *Listeria monocytogenes* (*n* = 4), *Shigella dysenteriae* (*n* = 2), *Staphylococcus caprae* (*n* = 4), *Staphylococcus gallinarum* (*n* = 2), *Streptococcus dysgalactiae* (Gr C) (*n* = 2), *Staphylococcus saprophyticus* (*n* = 5), *Streptococcus* gr C (*n* = 2), *Streptococcus salivarius* (*n* = 2), *Streptococcus sobrinus* (*n* = 2). On the other hand, 38 species were isolated only since the beginning of the COVID-19 pandemic (Supplementary Table S7). Even in this case, only one strain, mostly from COVID+ patients, was isolated for several (29) of these species (Supplementary Table S7). Conversely, several strains were isolated for the species *Enterobacter asburiae*, *Serratia plymuthica*, *Staphylococcus cohnii* spp. *urealyticum* (*n* = 2), *Staphylococcus sciuri*, *Staphylococcus simulans*, *Streptococcus dysgalactiae* spp. *equisimilis* (*n* = 3), *Staphylococcus lugdunensis* and *Citrobacter farmeri* (respectively *n* = 7 and *n* = 6) (Supplementary Table S7).

**Figure 4:**
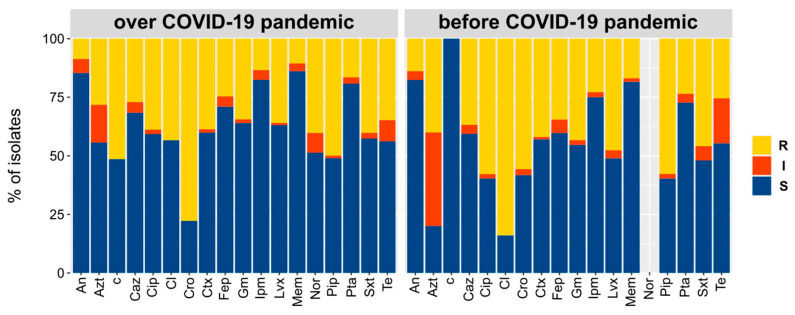
Percentages of strains, isolated during (**left**) and before (**right**) the COVID-19 pandemic, resistant to the tested antibiotics. An = amikacin, Azt = aztreonam, Fep = cefepime, Ctx = cefotaxime, Caz = ceftazidime, Cro = ceftriaxone, Ipm = imipenem, Lvx = levofloxacin, Mem = meropenem, Nor = norfloxacin, Pip = piperacillin, Pta = piperacillin-tazobactam, Te = tetracycline, Sxt = trimethoprim/sulfamethoxazole, Gm = gentamicin, Cl = colistin, Cip = ciprofloxacin, c = chloramphenicol. The test aimed at evaluating susceptibility to norfloxacin was introduced after gathering data relative to “before COVID-19 pandemic”.

Overall, the response of isolates to the tested antibiotics did not change between before and during the COVID-19 pandemic (Figure 4, Chi-square test *p* > 0.05). The same situation was observed when comparing the susceptibilities to the tested antibiotic between co-specific strains isolated before and during the pandemic (Chi-square test *p* > 0.05), indicating that either the pandemic has not promoted the insurgence of antibiotic resistance or that the increase is not yet observable.

## 4. Discussion

Our dataset of bacterial strains isolated from clinical samples over one year from the onset of the COVID-19 pandemic provided a balanced representation of bacterial infections occurring at various body sites and in various clinical areas, both in COVID+ and COVID− patients. In fact, we did not observe any significant difference between COVID+ and COVID− isolates in the distribution of isolates among clinical areas or isolation sources. Conversely, we could observe that multiple strains from different species are more frequently isolated from COVID+ patients than from COVID− patients, also corroborated by a significantly higher number of species associated with COVID+ patients than with COVID− patients. This observation may be ascribed to the systemic frailty of patients suffering from the viral infection or to the prolonged utilization of invasive life support machines, which may promote the onset of microbial infections [29]. It is not possible to determine which of the two hypotheses prevails because multiple strains were more frequently isolated from specimens sampled from hospitalized patients than from ER patients, hence suggesting that single-strain infections are usually community-acquired and multiple-strains infection are more likely to be acquired over prolonged hospitalization periods, with the latter being commonly subjected to the use of ventilators or other invasive procedures and often suffering from pathologies resulting in a general debility. An alternative cause of the observation of multiple strains isolated from non-ER patients could be ascribed to the fact that, whereas ER patients were sampled only once, samples from non-ER clinical areas were mostly collected on multiple occasions (over the period of hospitalization of the patient). In fact, we found that the number of isolates per patient in the ER was significantly lower than the number of isolates per patient in medicine and surgery. However, the fact that multiple strains were isolated from COVID+ patients cannot be entirely ascribed to the repeated sampling of these patients over ER-patients because we did not observe a significant difference between COVID+ and COVID− patients in the ER. It has to be considered that, despite the identification of bacterial strains as potentially causative of the infection is carried out by taking into consideration the entire clinical profile and symptoms of the patient, some of the isolates included in our dataset, especially those found in co-occurrence with other strains, may be commensal, rather than the pathogens responsible for the infection. However, considering that the main aim of this study was to assess the impact of the COVID-19 pandemic on the spread of antimicrobial resistance, the results should not be affected by the inclusion of commensal strains.

To note, the number of bacterial isolates from COVID+ patients was correlated with the number of new COVID-19 cases monitored at the national level, suggesting a high chance of bacterial infection co-occurrence. In correspondence with the peak in the number of new COVID-19 cases the government imposed general lockdown measures (limiting socialization) and hospitalization for planned and delayable procedures was postponed to redirect the activity of sanitary workers toward the cure and care of COVID-19 patients (thus reducing the chances of occurrence of nosocomial infections). Hence, we expected to find a negative correlation between the number of bacterial isolates from COVID− patients and the number of new cases. Conversely, this hypothesis was not statistically supported, possibly indicating that the limitations imposed for the sanitary emergency did contain the spread of the virus, but not the insurgence of bacterial infections. The observation of the correlation between the number of bacterial isolates and the number of new COVID-19 cases at the national level can be an indication of the fact that all the COVID-19 patients have the same chance of acquiring a bacterial infection, (hence if the number of COVID-19 cases increases then also the number of bacterial isolates does) and that the number of new COVID-19 patients hospitalized at the hospital under investigation in this study has the same trend as the number of new COVID-19 cases at the national level. Whereas we could not evaluate the validity of the first point, as it can be evaluated only through a multi-center survey; however, we did confirm the latter through the observation of a positive correlation between the number of new COVID-19 cases at the national and provincial (Turin) levels (Pearson correlation r = 0.677 *p* < 2.2 × 10^−16^, Supplementary Figure S7).

Not surprisingly, considering the overall debilitation of COVID+ patients, a large part of the species identified only among the isolates from COVID+ patients are commonly associated with infections in immuno-compromised patients (*Burkholderia cepacia* and *Citrobacter braakii*) or nosocomial infections (*Sphingomonas paucimobilis*) [30,31,32]. To note, the observation that *Klebsiella pneumoniae* was isolated more frequently from COVID+ patients than from COVID− patients, possibly associated with the use of invasive devices widely used in the treatment of COVID-19 (ventilator and urinary catheters), may support the current dread of increase of nosocomial infections and AMR as an indirect consequence of the COVID-19 pandemic. In fact, this species is among the most dreadful current causes of healthcare-associated infections including pneumonia, urinary tract infections, and bloodstream infections and is also frequently associated with multi-drug resistance [33]. On the other hand, the observation of *Acinetobacter baumannii/haemolyticus* being more abundant among the COVID+ isolates than among the COVID− ones is not surprising, as this species is known to cause ventilator-associated and bloodstream infections in critically ill patients [34], both conditions obviously present in COVID+ patients. Conversely, *Escherichia coli*, one of the most frequent causes of common bacterial infections in humans [35] and the most frequently isolated species in our dataset, was also more frequently isolated from COVID− than from COVID+ patients. Furthermore, the fact that *E. coli* was the species most likely represented among the urine isolates in COVID− patients, as commonly occurring in urinary tract infections [36] and not in COVID+ patients suggests that in the presence of SARS-CoV-2 infections, this opportunistic pathogen can equally infect various body sites.

Our dataset seems to support the predicted impact of the COVID-19 on the spread of AMR infections: the fraction of microorganisms resistant to every tested antibiotic is higher in COVID+ than in COVID− isolates. Furthermore, COVID+ isolates show higher resistance to amikacin and gentamicin (aminoglycosides), aztreonam (monobactam), levofloxacin (third-generation fluoroquinolone), meropenem (beta-lactam). All the above-mentioned antibiotics are used as the first line therapeutic agents for the management of severe pneumonia, and the diffusion of resistance toward them limits their efficacy. Moreover, the possibility of exploiting the antiviral activity of these antibiotics against SARS-CoV-2 was evaluated, but the ability of fluoroquinolones (such as levofloxacin) to suppress SARS-CoV-2 and MERS-CoV replication in cultured cells has been proven limited [11], the effectiveness of aminoglycosides (such as gentamicin and amikacin) against SARS-CoV-2 due to production of retrocyclins [9], and the binding of meropenem to SARS-CoV-2 protease [10] still need to be demonstrated. Hence, it is reasonable to assume that the antibiotics toward which COVID+ isolates showed a high resistance had been administered as part of the therapy, thus promoting the selection of resistant strains. To further confirm these findings, future investigations will have to consider clinical and demographic characteristics of the patients infected by bacteria, hence assessing the impact of variables such as age, gender, presence of comorbidities, and clinical treatments on the insurgence of bacterial infection and, eventually, on the occurrence of antibacterial resistance.

It is worth noting that neither COVID+ nor COVID− isolates showed significantly higher resistance to trimethoprim/sulfamethoxazole, indicated among the drugs to be administered to COVID+ patients, nor to ceftriaxone, a cephalosporin indicated for COVID-19 infection domestic treatment in the presence of a reasonable suspicion of concomitant bacterial or viral infection over the early phases of the pandemic [12]. However, our study lacks the population of COVID+ patients who have not been hospitalized, due to government regulations recommending limiting access to hospitals and treating patients at home whenever possible. In this regard, therefore, we cannot exclude that the lack of observation of resistance to antibiotics used mainly outside the hospital is due to the fact that we have not analyzed the population exposed to those antibiotics. It is also worth noting that the significantly higher susceptibility of COVID− isolates to cefepime, cefotaxime, ceftazidime, imipenem, piperacillin, and piperacillin-tazobactam may indicate that COVID+ isolates show more frequently an intermediate response or are resistant to these antibiotics, suggesting a trend toward the increase of AMR against them. Even more worryingly, more COVID+ isolates than COVID− isolates were resistant to every tested antibiotic, further suggesting that the COVID-19 infection has an impact on the insurgence or selection of multi-resistant strains. This observation seems to be particularly relevant for the *Burkholderia cepacia*, *Corynebacterium renale*, *Corynebacterium jeikeium*, *Corynebacterium. matruchotii*, *Corynebacterium amycolatum*, *Enterococcus avium*, and *Enterococcus faecium* species, whose strains isolated from COVID+ patients showed high levels of multiple antibiotic resistance. However, the comparison of strains isolated before and over the course of the COVID-19 pandemic provided a different and hence slightly reassuring portrait: the level of antibiotic resistance against the tested antibiotic is not significantly different among the two sets. Hence overall, the delayed treatments, and the diagnostic and surgical errors observed over the pandemic [37] have not obviously (or yet) impacted on the spread of bacterial infection and on the insurgence of resistances. Nevertheless, this observation has to be taken with caution. In fact, an increase in antibiotic resistance may be occurring, but it may not be perceivable yet.

Supporting the hypothesis that a change in microbial infections is occurring in association with the COVID-19 pandemic is the observation of differences between the microbial species identified before and during the pandemic. The fact that some species were isolated only before or over the COVID-19 pandemic may be ascribed to different social behaviors over the pandemic or, in the case of species more frequently isolated from COVID+ patients, to the COVID-19 infection *per se* (or to the adopted therapy). It is interesting to observe that, despite the analytical procedure for the identification of *Staphylococcus lugdunensis* has been recently improved, such an improvement was achieved before the acquisition of the dataset relative to strains isolated before the COVID-19 pandemic [38]. Hence, the lack of observation of this species before the pandemic must not be ascribed to the incapability of identifying this species.

## 5. Conclusions

The key points emerging from our study are highlighted hereafter:

1. No significant differences were observed between COVID+ and COVID− in the distribution of isolates among clinical areas or isolation sources;
2. Conversely, multiple strains from different species are more frequently isolated from COVID+ patients than from COVID− patients, and a significantly higher number of species was associated with COVID+ patients than with COVID− patients;
3. The number of bacterial isolates from COVID+ patients was correlated with the number of new COVID-19 cases monitored at the national level;
4. Several species identified only among COVID+ patients are commonly associated with infections in immuno-compromised patients or nosocomial infections. *Klebsiella pneumoniae* and *Acinetobacter baumannii/haemolyticus* were more abundant among COVID+ isolates;
5. The fraction of microorganisms resistant to every tested antibiotic is higher in COVID+ than in COVID− isolates. Furthermore, COVID+ isolates show higher resistance to several antibiotics used as the first line therapeutic agents for the management of severe pneumonia;
6. Resistance to trimethoprim/sulfamethoxazole was equally distributed between COVID+ and COVID− isolates;
7. More COVID+ than COVID− isolates were resistant to every tested antibiotic.

The COVID-19 pandemic has widely impacted not only the common habits of us all but also the profile of microbial infections and antimicrobial resistance. The changes in our social behaviors may have contributed to differentiating the species associated with infections before and during the current pandemic, but the predicted AMR, as a consequence of the administration of broad-spectrum antibiotics to COVID+ patients is not observable yet. However, the higher frequency, among COVID+ isolates, of strains resistant to every tested antibiotic seems to indicate that the AMR increase is actually occurring, hence further calling for the definition of alternative treatments of COVID-19 infections, eventually encompassing antibiotics accurately selected if necessary and upon evaluation of the pathogen’s antibiogram.

## Data Availability

Data is contained within the article or supplementary material.

## Supplementary Materials

The following are available online at www.mdpi.com/article/10.3390/biology10090822/s1. Supplementary Figure S1: Distribution of samples between COVID+ and COVID− patients. Supplementary Figure S2: Distribution of samples among clinical areas. Supplementary Figure S3: Number of strains isolated from COVID+ or COVID− patients hospitalized in the clinical areas under investigation. Supplementary Figure S4: Distribution of isolation sources among COVID+ and COVID− patients. Supplementary Figure S5: Alpha diversities of samples. Supplementary Figure S6: Correlations between the percentages of COVID+ and COVID− isolates resistances to the tested antibiotics and the number of new COVID-19 cases detected at the national level. Supplementary Figure S7: Number of new COVID-19 cases monitored at the national and provincial (Turin) levels. Other supplementary information associated with this study (provided as separated files): Supplementary Table S1: Details on the clinical bacterial isolates analyzed over this study. Supplementary Table S2: Details on the lists of antibiotics tested on the isolated species. Supplementary Table S3: Bacterial species isolated only from either COVID+ or COVID− patients. Supplementary Table S4: Wilcoxon-Mann-Whitney p values of the comparison between alpha diversities calculated for isolation sources. Supplementary Table S5: Distribution of bacterial isolates among COVID+ and COVID− patients and details on taxa. Supplementary Table S6: Distribution of resistances and susceptibilities among isolates. Supplementary Table S7: Species isolated before and/or during the COVID-19 pandemic.
