# Supplementary material for: Profile of Bacterial Infections in COVID-19 Patients: Antimicrobial Resistance in the Time of SARS-CoV-2
Source: Biology (Basel). 2021 Aug 24;10(9):822. doi: 10.3390/biology10090822 (PMC8467430; doi:10.3390/biology10090822)

Supplementary information for

Profile of bacterial infections in COVID-19 patients: antimicrobial resistance in the time of SARS-CoV-2

Irene Stefanini[1], Giuseppe De Renzi[2], Elisa Foddai[2], Elisa Cordani[2], Barbara Mognetti[1,*]

[1] Department of Life Sciences and Systems Biology, University of Turin, Via Accademia Albertina 13, 10123 Turin, Italy.

[2] SCDO Laboratory of Clinical Pathology and Microbiology, San Luigi Gonzaga University Hospital, Regione Gonzole 10, Orbassano, 10043 Turin, Italy.

This pdf includes:
Supplementary Figure S1: Distribution of samples between COVID+ and COVID- patients.
Supplementary Figure S2: Distribution of samples among clinical areas.
Supplementary Figure S3: Number of strains isolated from COVID+ or COVID- patients hospitalized in the clinical areas under investigation.
Supplementary Figure S4: Distribution of isolation sources among COVID+ and COVID-patients.
Supplementary Figure S5: Alpha diversities of samples.
Supplementary Figure S6: Correlations between the percentages of COVID+ and COVID-isolates resistances to the tested antibiotics and the number of new COVID cases detected at the national level.
Supplementary Figure S7: Number of new COVID-19 cases monitored at the national and provincial (Turin) levels.

Other supplementary information associated with this study (provided as separated files):
Supplementary Table S1: Details on the clinical bacterial isolates analyzed over this study.
Supplementary Table S2: Details on the lists of antibiotics tested on the isolated species.
Supplementary Table S3: Bacterial species isolated only from either COVID+ or COVID-patients.
Supplementary Table S4: Wilcoxon-Mann-Whitney p values of the comparison between alpha diversities calculated for isolation sources.
Supplementary Table S5: Distribution of bacterial isolates among COVID+ and COVID-patients and details on taxa.
Supplementary Table S6: Distribution of resistances and susceptibilities among isolates.
Supplementary Table S7: Species isolated only before or over the COVID-19 pandemic.

Supplementary Figure S1

Distribution of samples between COVID+ and COVID- patients. Distribution of the number of isolates per specimen from COVID+ and COVID- patients. Gray bars and area= samples from COVID- patients, black bars and area= samples from COVID+ patients.

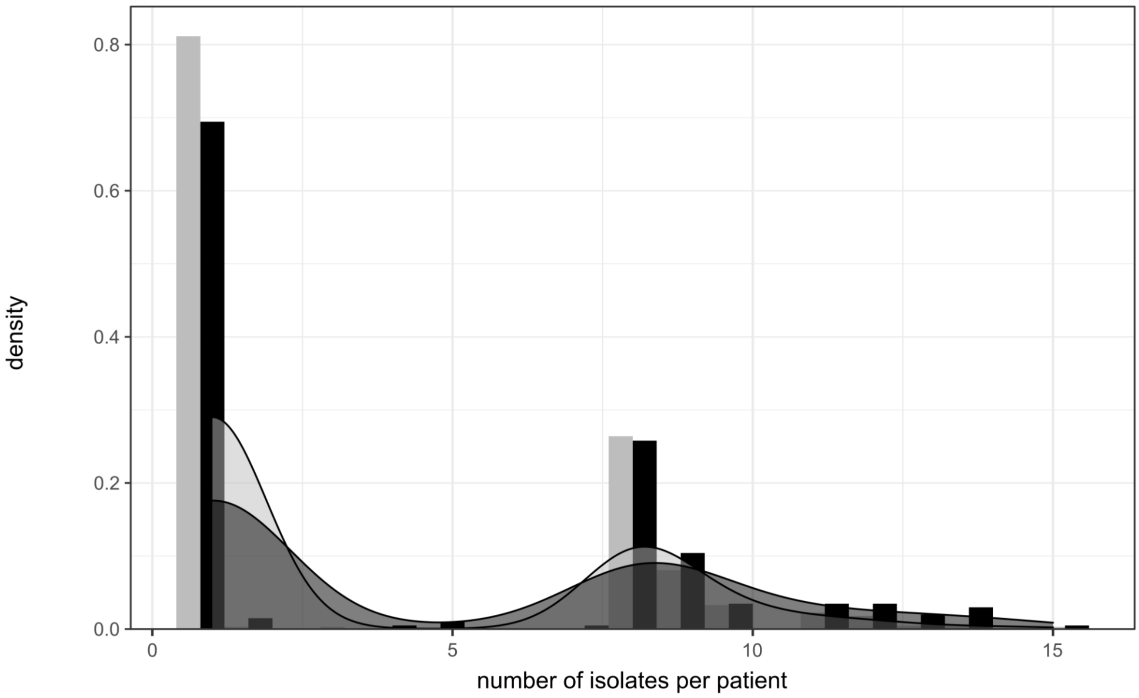

Supplementary Figure S2

Distribution of patients and isolates among clinical areas. **a)** Venn diagram reporting the distribution of patients among clinical areas and highlighting the patients that were hospitalized in multiple clinical areas. The numbers in the diagram areas indicate the total number of patients relative to the corresponding group (upper number) and the number of COVID+ (lower black Italic number) or COVID- patients (lower gray Italic number) relative to the corresponding group. Distribution of **b**) patients (and relative isolates) positive for COVID at the time of sampling and **c**) patients (and relative isolates) negative for COVID at the time of sampling (right). The outer pie charts report the information on patients, the inner pie charts report the information on isolates. Patients hospitalized in multiple clinical areas were counted in both clinical areas.

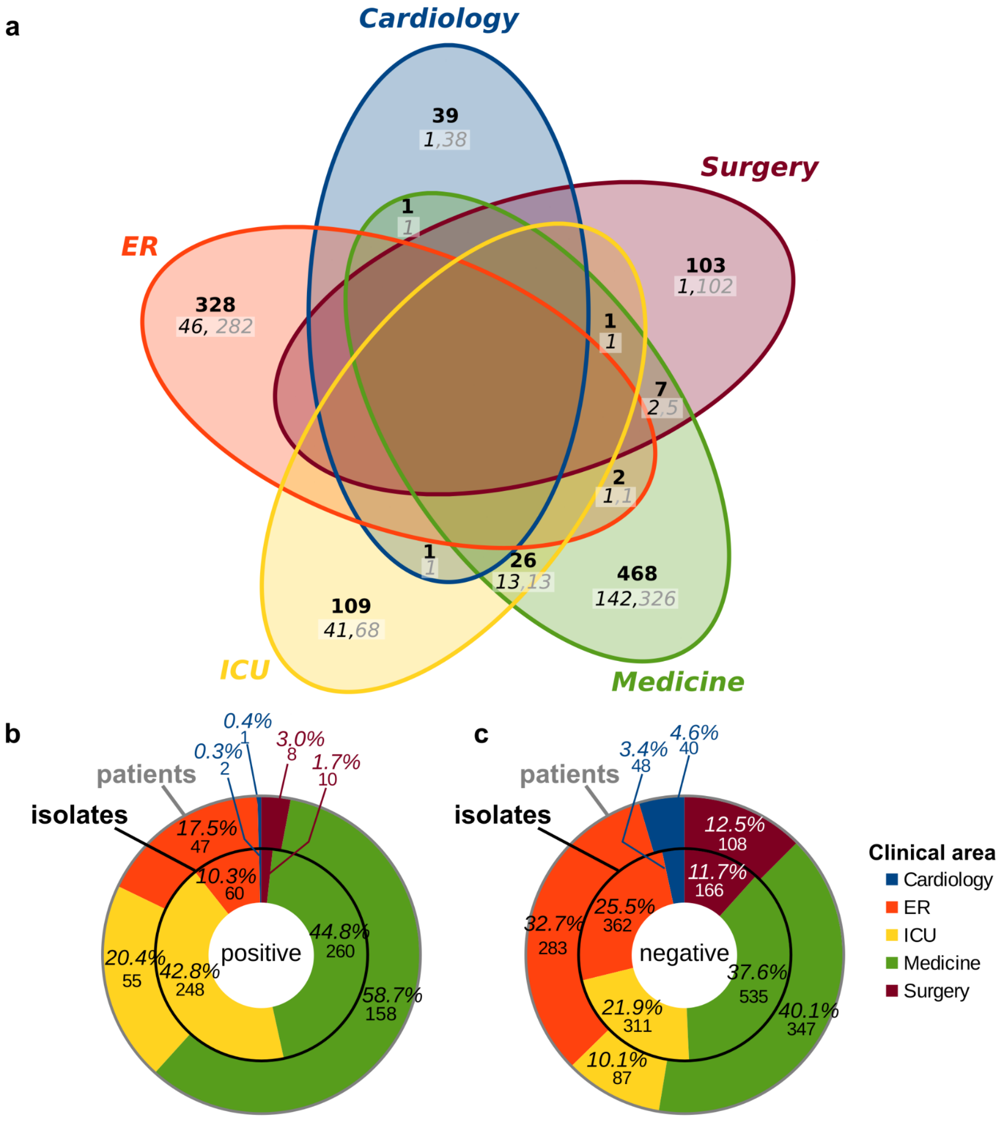

Supplementary Figure S3

Number of strains isolated from COVID+ or COVID- patients hospitalized in the clinical areas under investigation. Each point indicates a single patient. The single COVID+ patient hospitalized in the cardiology area was not considered for this analysis as its singularity would affect the statistical test.

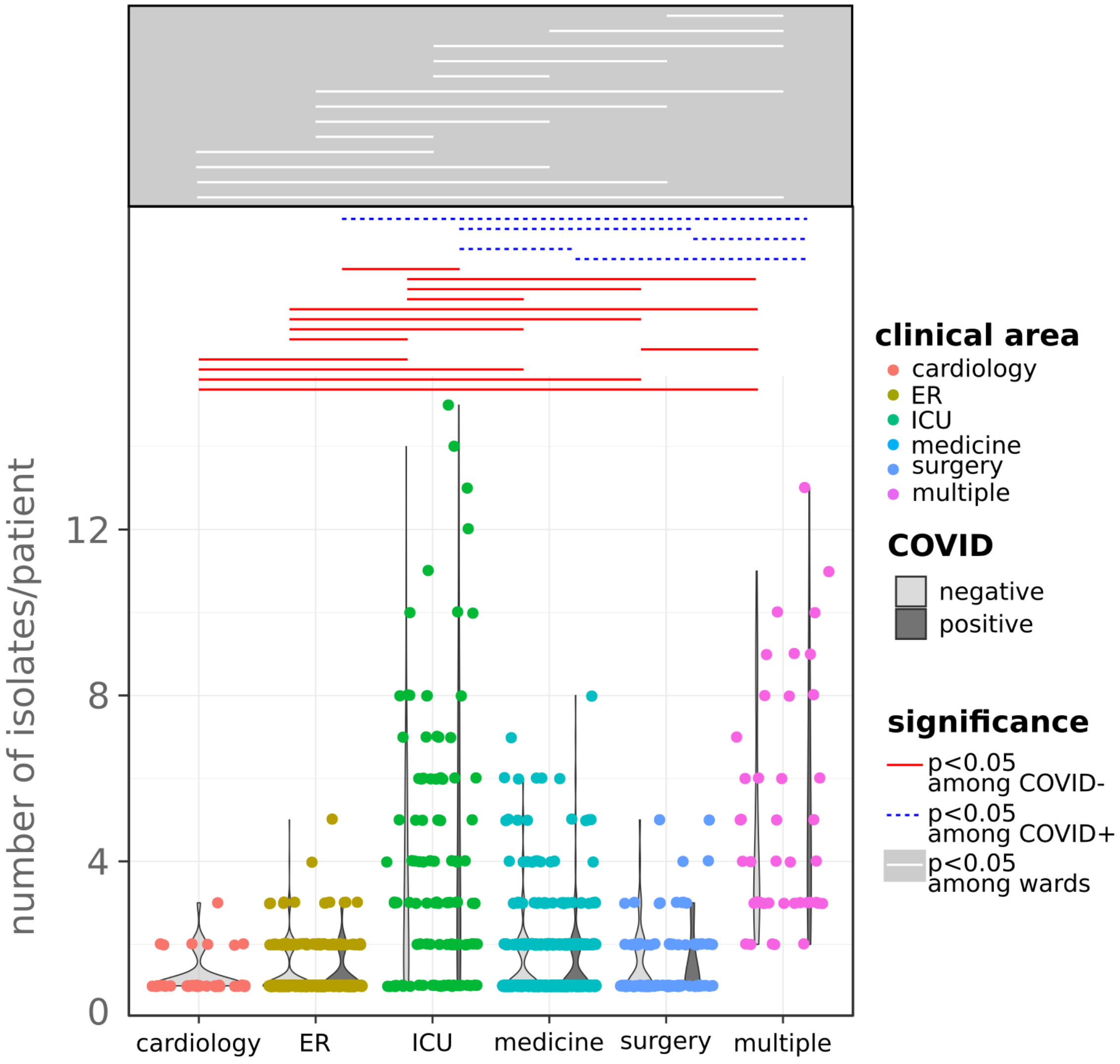

Supplementary Figure S4

Distribution of isolation sources among COVID+ and COVID- patients.

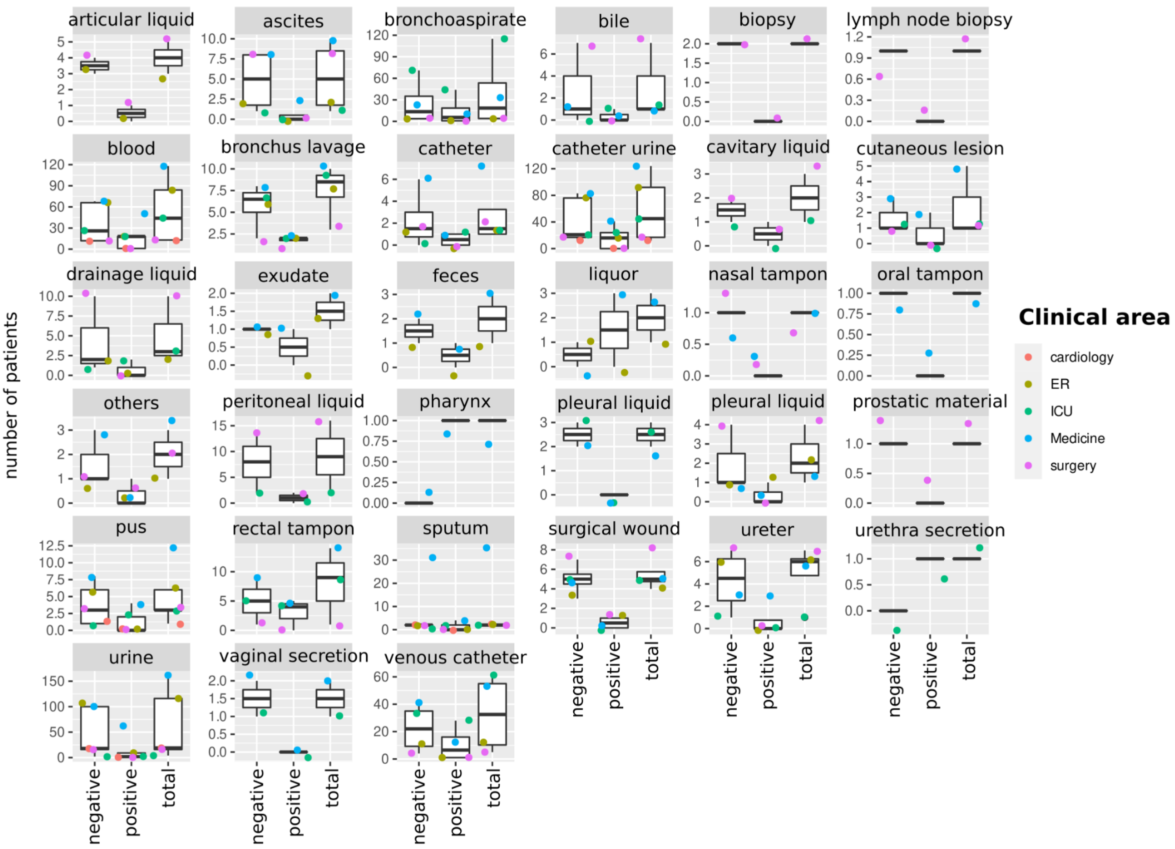

Supplementary Figure S5

Alpha diversities of samples. Strains were grouped according to the month and year of isolation, the isolation source, the clinical area, and the COVID status of the patient. comparison of alpha diversities among groups according to **a)** the COVID status of the patient, **b)** the clinical area and **c)** the specimen source of the isolation. Horizontal black lines above the violin plots in panels **a** and **b** indicate significant differences among the connected groups of samples (Wilcoxon-Mann-Whitney p<0.05). Wilcoxon-Mann-Whitney p values resulting from the comparisons among groups of panel **c)** are reported in **Supplementary Table S3**.

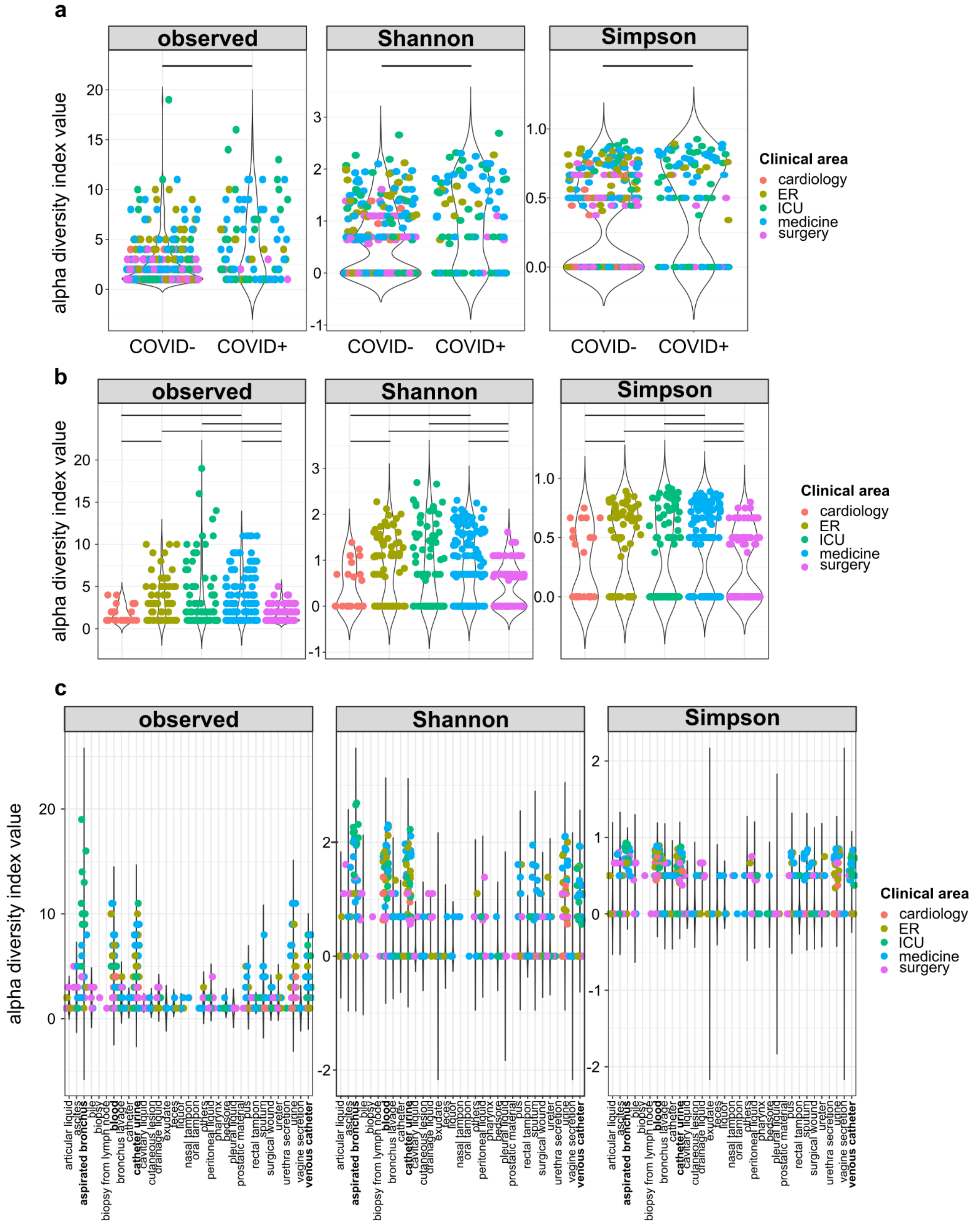

Supplementary Figure S6

Correlations between the percentages of COVID+ and COVID- isolates resistances to the tested antibiotics and the number of new COVID cases detected at the national level. The text under the antibiotic label indicates the results of the Pearson correlation test, for COVID+ isolates (+, upper text) and for COVID- isolates (-, lower text). The asterisks indicate a significant correlation between the indicated group of isolates (COVID+ or COVID-) and the number of new COVID cases identified at the national level ($p<0.05$).

An= amikacin, Azt= aztreonam, Fep= cefepime, Ctx= cefotaxime, Caz= ceftazidime, Cro= ceftriaxone, Ipm= imipenem, Lvx= levofloxacin, Mem= meropenem, Nor= norfloxacin, Pip= piperacillin, Pta= piperacillin-tazobactam, Te= tetracycline, Sxt= trimethoprim/sulfamethoxazole, Gm= gentamicin, Cl= colistin, Cip= ciprofloxacin, c= chloramphenicol.

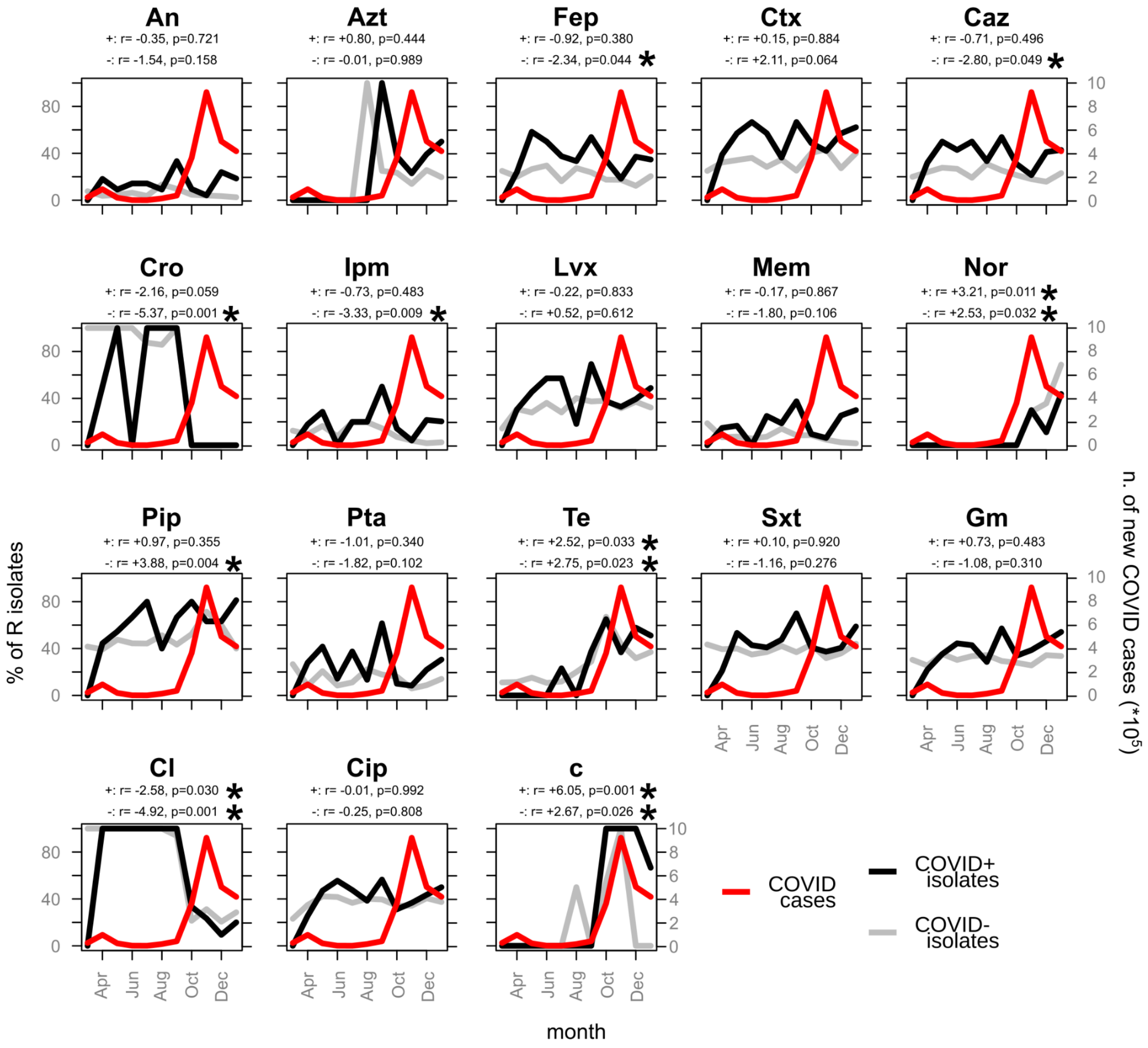

Supplementary Figure S7

Number of new COVID-19 cases monitored at the national and provincial (Turin) levels.

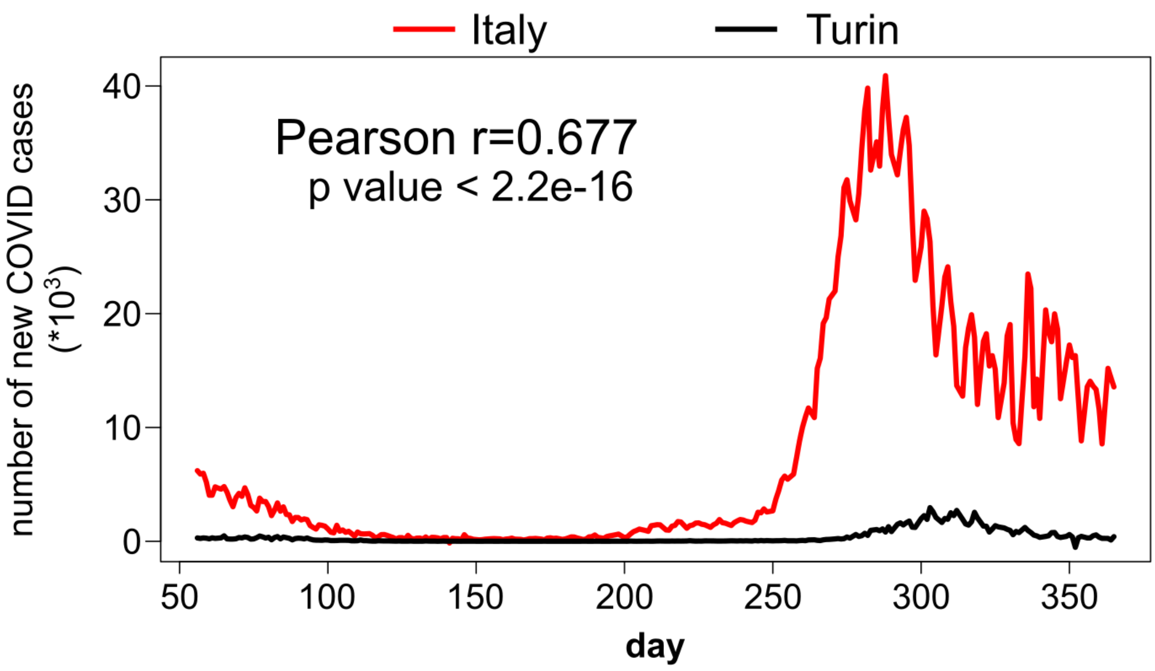

Supplement: Supplementary file 1 [file biology-10-00822-s001.zip › Supplementary_information.pdf]
